# Testing the effect of stimulus onset asynchrony on auditory attention using the attention network test

**DOI:** 10.3389/fpsyg.2026.1726700

**Published:** 2026-01-21

**Authors:** Tianfang Han, Arianna N. LaCroix

**Affiliations:** 1 Department of Psychology and Communication, University of Idaho, Moscow, ID, United States; 2 Department of Speech, Language, and Hearing Sciences, Purdue University, West Lafayette, IN, United States

**Keywords:** alerting, attentional networks, auditory attention, executive control, foreperiod, orienting, stimulus onset asynchrony

## Abstract

**Introduction:**

The Attention Network Test (ANT) is a widely used paradigm for assessing the efficiency of attentional subsystems. Although most ANT implementations rely on visual cues and stimuli, extending the ANT to the auditory domain is important for advancing theories of modality-specific attention and for enabling assessment in populations with visual impairments.

**Methods:**

The present study adapted an auditory version of the ANT to examine how stimulus onset asynchrony (SOA) modulates alerting, orienting, and executive control attention. Participants completed a duration discrimination task in which they judged whether the first of two tones (the target) was short or long while ignoring the second tone (the flanker). Trials were preceded by one of three cues: no cue, an alerting cue (pink noise), or an orienting cue (pink noise paired with a target-matching pure tone). SOAs were either 400 ms or 900 ms.

**Results:**

The task elicited robust executive control effects, with slower and less accurate responses on incongruent compared to congruent trials, as well as reliable orienting effects, with faster responses following orienting versus neutral cues. In contrast, the alerting effect was not significant. However, alerting interacted with executive control: congruency effects were reduced following alerting cues, a pattern opposite to that observed in the visual ANT but consistent with prior auditory work. SOA further modulated performance, with evidence that alerting cues were more effective at shorter intervals, whereas orienting cues exerted greater influence at longer intervals.

**Discussion:**

These findings support the feasibility and promise of an auditory ANT while highlighting important temporal constraints on auditory attention. Future work will be needed to refine the task to better capture the dynamic interplay among attentional networks in the auditory domain.

## 1 Introduction

Attention is a multifaceted cognitive system that enables the selection and prioritization of sensory information to support perception and goal-directed behavior. According to the influential model by Posner and Petersen (1990, 2012), attention comprises three anatomically and functionally distinct networks: alerting, which maintains a state of readiness to respond; orienting, which directs attention to a particular location or stimulus feature; and executive control, which resolves conflict among competing responses. These networks interact dynamically and can be dissociated experimentally using a cued-flanker task known as the Attention Network Test (ANT) (Fan et al., 2002). Performance on the ANT is typically faster when participants receive informative cues about flanker timing (alerting) or location (orienting), or when the target is flanked by congruent stimuli (executive control; Fan et al., 2002).

The ANT and its variants have been used widely to study attention across diverse populations, including bilinguals (Costa et al., 2008) and clinical groups such as individuals with anxiety (Pacheco-Unguetti et al., 2010), ADHD (Johnson et al., 2008), borderline personality disorder (Posner et al., 2002), deafness (Dye et al., 2007), aphasia (Dash et al., 2020; LaCroix et al., 2020; Mohapatra and Dash, 2023), and schizophrenia (Wang et al., 2005). Its widespread use is likely attributed to its strong theoretical grounding, reliability in measuring all three attention networks, ability to address interactions between networks, and ease of use, including its short duration. However, the ANT variants largely use visual cues and flanker targets (e.g., ANT-I by Callejas et al., 2005, ANT-R by Fan et al., 2009, AttentionTrip by Klein et al., 2017; de Souza Almeida et al., 2021), which precludes them from being used with individuals with visual impairments, while also constraining their relevance to auditory attention research.

Efforts have been made to develop auditory versions of the ANT, beginning with Roberts et al. (2006) and followed by the ANT-AS developed by Spagna et al. (2015). In both tasks, participants responded to the pitch of a monoaurally presented auditory target—human voice in Roberts et al.’s task and a pure tone in the ANT-AS. The target’s pitch was either congruent or incongruent with a distractor stimulus’ pitch, enabling assessment of executive control based on performance differences across trial types. Alerting and orienting were assessed by comparing responses to binaural (alerting) or monaural (orienting) auditory cues, such as clicks or white noise, to no cue and neutral spatial cues, respectively. Both tasks elicited robust congruency effects that correlated with those observed in the original visual ANT, supporting the domain-general nature of executive control. They also produced clear alerting effects, with faster responses following binaural cues; however, these alerting effects did not correlate across modalities, suggesting alerting attention may use modality-specific processes. In contrast, monaural spatial cues failed to produce reliable orienting effects in either task, potentially due to the inherent difficulty of cueing spatial attention through the auditory system—a finding later replicated by Johnston et al. (2019), who also observed no spatial auditory orienting response.

Spagna et al. (2015) also introduced the ANT-AF, which uses a duration discrimination task (rather than pitch) to assess executive control, and frequency cues (rather than clicks) to assess alerting and orienting. The ANT-AF produced similar findings to the ANT-AS for alerting and executive control. However, unlike the ANT-AS, the ANT-AF successfully elicited an orienting effect: valid frequency cues—those that matched the frequency of the target tone—enhanced response speed compared to frequency neutral cues. The authors suggested that frequency information may be better suited for cueing the auditory orienting system because the cochlea’s tonotopic organization allows frequency to be extracted directly from the stimulus, whereas spatial information must be computed. Like alerting attention, orienting attention did not correlate across the auditory and visual modalities, suggesting that orienting may also operate in a modality-specific manner. These few studies raise important questions about whether visual attention tasks offer meaningful insight into auditory attention, and whether the well-established patterns from the visual ANT generalize to the auditory domain.

One key parameter differentiating auditory and visual versions of the ANT is the interval between cue onset and target onset, known as stimulus onset asynchrony (SOA) in the alertness literature and foreperiod duration more broadly. In visual paradigms, attentional performance improves with shorter (<500 ms) and more predictable (or fixed duration) SOAs (de Souza Almeida et al., 2021; Los, 2010; Niemi and Naatanen, 1981; Posner and Petersen, 1990; Sturm and Willmes, 2001). In contrast, auditory ANTs have adopted longer SOAs (600–900 ms), based on the assumption that auditory cueing requires additional processing time to fully develop, and also to avoid temporal overlap between the cue and target (Roberts et al., 2006; Spagna et al., 2015). This raises the possibility that phasic arousal operates on a broader temporal window in the auditory modality, although this has not been systematically tested. Even less is known about how SOA influences auditory orienting. Johnston et al. (2019), for example, found that spatial auditory orienting effects were only observed with short SOAs (150 ms), while longer cue-target intervals (>450 ms) failed to elicit such effects. Yet, it remains unclear whether SOA similarly influences non-spatial auditory orienting, particularly in tasks that use frequency-based cues.

The current study explored the effect of SOA on the attentional subsystems using an auditory version of the ANT. A duration discrimination task was used to measure executive control attention and noise bursts and frequency cues were used to measure alerting and orienting attention. We hypothesized that the alerting effect (faster responses on noise burst trials compared to no cue trials) would emerge at shorter SOAs (e.g., 400 ms) and that the orienting effect (faster responses on frequency cues compared to noise bursts) would emerge at longer SOAs (e.g., 900 ms). We additionally expected executive control effects (faster responses on congruent than incongruent trials) to emerge at both SOAs.

## 2 Method

### 2.1 Participants

Forty-seven participants took part in this study. One participant was excluded due to being diagnosed with an attention disorder, and a second participant was excluded due to a history of mild traumatic brain injury. Thirteen participants were excluded due to having <70% accuracy in at least one condition. The final sample included 32 participants (22 females) ranging in age from 18 to 33 years (*M* = 20.63, *SD* = 3.08). On average, participants had 13.45 years of education (*SD* = 1.54), and all were right-handed, English speakers, with normal or corrected-to-normal vision, and hearing thresholds within the normal range (i.e., pure tone averages < 25 db at 500, 1,000, and 2,000 Hz). An *a priori* power analysis, using a medium effect size derived from the cue x interstimulus interval interactions reported in Johnston et al. (2019), indicated that a minimum of 24 participants was required to achieve 0.80 power. The reported effect size (η^2^_p_ = ~0.06) was converted to Cohen’s f (~0.25) for use in G*Power, assuming a correlation of 0.50 among repeated measures. This calculation was designed to ensure adequate power for detecting the two-way interaction in both reaction times and error rates, which were the primary outcomes of interest. Therefore, our final sample size of 32 was considered adequate for the planned analyses. Participants provided written informed consent and were monetarily compensated for their participation. Purdue University’s Institutional Review Board approved all study procedures (IRB-2022-1324).

### 2.2 Attention network test

The task was administered using E-Prime 3.0 on a Dell Latitude 5330 laptop. A fixation cross remained on the screen throughout to help participants maintain visual focus. Auditory stimuli were presented binaurally through noise-canceling headphones at a comfortable, individually adjusted volume. Participants were informed that the testing session would last 30–40 min. They were instructed that on each trial they would hear two pure tones and must decide whether the first tone (the target) was short or long in duration, while ignoring the duration of the second tone (the flanker). Participants responded using their left hand on the “z” key (labeled “s”) and “x” key (labeled “l”), which were selected because the task is designed for future use with left hemisphere stroke survivors, many of whom experience right-sided weakness or hemiparesis. Using left hand responses ensures consistency with our broader research program and minimizes motor-related variability across populations. The “z” and “x” keys were chosen because they are adjacent and allow for minimal finger movement, with short tones mapped to “z” (labeled “s”) and long tones to “x” (labeled “l”), following a left-to-right spatial continuum. Speed and accuracy were equally emphasized, and both reaction time and accuracy were recorded on each trial.

**Figure 1:**
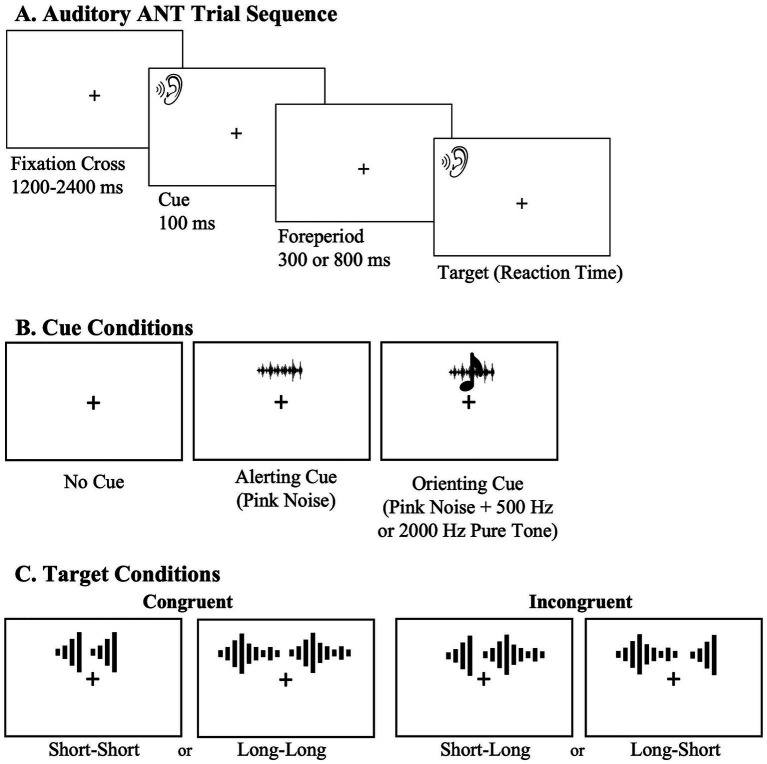
Visual schematic of the trial sequence for the auditory ANT **(A)**, cue conditions **(B)**, and target conditions **(C)**. The central fixation cross was presented for the total duration of the task. The ear image represents when an auditory stimulus was presented. All auditory stimuli were presented binaurally.

The experiment began with a general introduction, followed by one example each of a short (150 ms) and long (450 ms) target tone, and a practice block of eight trials. The main task consisted of two blocks that differed only in SOA: 400 ms or 900 ms. Block order was counterbalanced across participants. Each block comprised 144 trials, evenly distributed across cue and target types. On each trial, a variable fixation interval (1,200–2,400 ms) was followed by a 100-ms cue, the foreperiod (300 or 800 ms), and then the target (Figure 1A).

The task included three cue conditions (Figure 1B). In the no-cue condition, participants heard 100 ms of silence, which provided no temporal or frequency information. In the alerting condition, participants heard 100 ms of pink noise, which increased temporal readiness by signaling the onset of the upcoming target. In the orienting condition, participants heard 100 ms of pink noise presented simultaneously with a pure tone that matched the frequency of the upcoming target tone. This frequency cue was intended to direct attention toward the relevant auditory channel in advance, thereby facilitating target discrimination. The efficiency of the alerting system was measured by comparing performance in the alerting condition, which provides temporal information, with the no-cue condition, which does not. The efficiency of the orienting system was measured by comparing the orienting condition, which provides frequency information, to the alerting condition, which does not.

Each target consisted of two pure tones separated by a 200-ms silent gap (Figure 1C). Short tones lasted 150 ms and long tones lasted 450 ms. Both tones were always presented at the same frequency, either 500 Hz or 2,000 Hz. The task contained two target conditions: trials were classified as congruent when the two tones had the same duration (short–short or long–long) and incongruent when the durations differed (short–long or long–short). The efficiency of the executive control system was measured by comparing performance on congruent and incongruent trials, with the latter requiring resolution of conflict between the target and flanker.

### 2.3 Statistical analyses

Prior to analysis, the first trial of each block (0.7%), trials with errors (2.3%), and trials with reaction times (RTs) more than two standard deviations above or below each participant’s mean for a given condition were excluded. To examine the impact of SOA on alerting attention, mean RTs and error percentages (EPs) were separately analyzed using repeated-measures ANOVAs with four within-subject factors: SOA (400 vs. 900 ms), cue type (alerting vs. no cue), previous congruency (congruent vs. incongruent), and current congruency (congruent vs. incongruent). We next examined the impact of SOA on orienting attention using the same repeated-measures ANOVA design, except that cue type contrasted the orienting and alerting conditions. All effects were tested at an α level of 0.05.

## 3 Results

The mean RTs and EPs, and corresponding standard deviations, for all experimental conditions, are presented in Table 1.

**Table 1:** Mean RT and EP separated by SOA, cue type, previous congruency, and current congruency.

SOA (ms)	Cue type	Previous congruency	Current congruency	RT (ms)	EP
400	No cue	Congruent	Congruent	1,152 (280)	2.2 (4.8)
			Incongruent	1,243 (339)	3.2 (5.9)
		Incongruent	Congruent	1,122 (237)	1.5 (4.8)
			Incongruent	1,231 (309)	2.1 (3.6)
	Alerting cue	Congruent	Congruent	1,168 (295)	1.9 (4.3)
			Incongruent	1,202 (303)	1.6 (4.6)
		Incongruent	Congruent	1,159 (281)	2.1 (4.1)
			Incongruent	1,223 (328)	4.2 (7.6)
	Orienting cue	Congruent	Congruent	1,143 (337)	0.8 (2.5)
			Incongruent	1,196 (377)	4.1 (6.7)
		Incongruent	Congruent	1,124 (284)	0.8 (3.0)
			Incongruent	1,189 (289)	2.6 (4.3)
900	No cue	Congruent	Congruent	1,171 (274)	1.2 (2.9)
			Incongruent	1,292 (390)	3.8 (7.0)
		Incongruent	Congruent	1,218 (284)	2.2 (5.3)
			Incongruent	1,315 (361)	2.7 (7.0)
	Alerting cue	Congruent	Congruent	1,192 (288)	0.6 (2.2)
			Incongruent	1,261 (338)	2.8 (4.9)
		Incongruent	Congruent	1,234 (347)	2.1 (5.5)
			Incongruent	1,256 (300)	3.2 (6.5)
	Orienting cue	Congruent	Congruent	1,170 (267)	1.4 (3.5)
			Incongruent	1,268 (377)	3.3 (6.7)
		Incongruent	Congruent	1,158 (264)	0.7 (3.0)
			Incongruent	1,232 (358)	4.1 (6.8)

Standard deviations are reported in parentheses.

### 3.1 Alerting ANOVA

#### 3.1.1 Reaction time

The full model results are reported in Table 2. The main effects of SOA and current congruency were significant. Responses were faster at the 400-ms SOA (1,188 ms) than the 900-ms SOA (1,242 ms), and on congruent (1,177 ms) compared to incongruent trials (1,253 ms). The two-way interaction between cue type and current congruency was also significant, showing that the executive control effect was smaller—though still significant *F*(1, 31) = 4.31, *p* = 0.046, η^2^_p_ = 0.12—following an alerting cue (48 ms) than a no cue (105 ms).

**Table 2:** Full model results for the alerting ANOVA.

Effect	Reaction time	Errors
SOA	***F*(1, 31) = 4.82, *p* = 0.036, η**^**2**^_**p**_ **= 0.13**	*F*(1, 31) < 0.01, *p* = 0.961, η^2^_p_ < 0.01
Cue type	*F*(1, 31) = 0.27, *p* = 0.607, η^2^_p_ = 0.01	*F*(1, 31) = 0.02, *p* = 0.893, η^2^_p_ < 0.01
Previous congruency	*F*(1, 31) = 0.61, *p* = 0.440, η^2^_p_ = 0.02	*F*(1, 31) = 0.31, *p* = 0.583, η^2^_p_ = 0.01
Current congruency	***F*(1, 31) = 16.45, *p* < 0.001, η**^**2**^_**p**_ **= 0.35**	***F*(1, 31) = 4.73, *p* = 0.037, η**^**2**^_**p**_ **= 0.13**
SOA × cue type	*F*(1, 31) = 0.39, *p* = 0.536, η^2^_p_ = 0.01	*F*(1, 31) = 0.35, *p* = 0.557, η^2^_p_ = 0.01
SOA × previous congruency	*F*(1, 31) = 1.39, *p* = 0.248, η^2^_p_ = 0.04	*F*(1, 31) = 0.06, *p* = 0.809, η^2^_p_ < 0.01
Cue type × previous congruency	*F*(1, 31) = 0.07, *p* = 0.790, η^2^_p_ < 0.01	***F*(1, 31) = 4.53, *p* = 0.041, η**^**2**^_**p**_ **= 0.13**
SOA × current congruency	*F*(1, 31) = 0.02, *p* = 0.901, η^2^_p_ < 0.01	*F*(1, 31) = 0.99, *p* = 0.328, η^2^_p_ = 0.03
Cue type × current congruency	***F*(1, 31) = 7.02, *p* = 0.013, η**^**2**^_**p**_ **= 0.19**	*F*(1, 31) = 0.02, *p* = 0.880, η^2^_p_ < 0.01
Previous congruency × current congruency	*F*(1, 31) = 0.03, *p* = 0.874, η^2^_p_ < 0.01	*F*(1, 31) = 0.12, *p* = 0.727, η^2^_p_ < 0.01
SOA × cue type × previous congruency	*F*(1, 31) = 0.92, *p* = 0.344, η^2^_p_ = 0.03	*F*(1, 31) = 1.43, *p* = 0.240, η^2^_p_ = 0.04
SOA × cue type × current congruency	*F*(1, 31) = 0.05, *p* = 0.823, η^2^_p_ < 0.01	*F*(1, 31) < 0.01, *p* = 0.981, η^2^_p_ < 0.01
SOA × previous congruency × current congruency	*F*(1, 31) = 1.25, *p* = 0.271, η^2^_p_ = 0.04	***F*(1, 31) = 4.30, *p* = 0.047, η**^**2**^_**p**_ **= 0.12**
Cue type × previous congruency × current congruency	*F*(1, 31) = 0.02, *p* = 0.887, η^2^_p_ < 0.01	*F*(1, 31) = 1.35, *p* = 0.255, η^2^_p_ = 0.04
SOA × cue type × previous congruency × current congruency	*F*(1, 31) = 0.20, *p* = 0.657, η^2^_p_ = 0.01	*F*(1, 31) = 0.31, *p* = 0.582, η^2^_p_ = 0.01

*Bolded results are significant at *p* < 0.05.

#### 3.1.2 Error percentage

The full model results are reported in Table 2. The main effect of current congruency was significant, with more errors occurring on incongruent (5.9%) than congruent trials (4.2%). The two-way interaction between cue type and previous congruency was also significant: when the previous trial was congruent, participants made more errors on no cue than alerting cue trials, but when the previous trial was incongruent participants made more errors on alerting cue than no cue trials. The three-way interaction between SOA, previous congruency, and current congruency was significant: at 900-ms SOA, participants made fewer errors when the congruency of the current trial matched that of the previous trial (e.g., congruent–congruent) and more errors when the congruencies were mismatched (e.g., incongruent–congruent). This pattern was present but less pronounced at the 400-ms SOA.

### 3.2 Orienting ANOVA

#### 3.2.1 Reaction time

The full model results are reported in Table 3. The main effects of cue type and current congruency were significant. Responses were faster following an orienting cue (1,185 ms) than an alerting cue (1,212 ms), and when trials were congruent (1,169 ms) versus incongruent (1,228 ms). The main effect of SOA trended toward significance (*p = 0*.09), with faster responses at the 400-ms SOA (1,176 ms) compared to the 900-ms SOA trials (1,221 ms).

**Table 3:** Results of the main ANOVA on orienting.

Effect	Reaction time	Errors
SOA	*F*(1, 31) = 2.22, *p* = 0.146, η^2^_p_ = 0.07	*F*(1, 31) < 0.01, *p* = 0.938, η^2^_p_ < 0.01
Cue type	***F*(1, 31) = 4.58, *p* = 0.040, η**^**2**^_**p**_ **= 0.13**	*F*(1, 31) = 0.05, *p* = 0.824, η^2^_p_ < 0.01
Previous congruency	*F*(1, 31) = 0.07, *p* = 0.787, η^2^_p_ < 0.01	*F*(1, 31) = 1.00, *p* = 0.325, η^2^_p_ = 0.03
Current congruency	***F*(1, 31) = 11.98, *p* = 0.002, η**^**2**^_**p**_ **= 0.28**	***F*(1, 31) = 7.74, *p* = 0.009, η**^**2**^_**p**_ **= 0.20**
SOA × cue type	*F*(1, 31) = 0.02, *p* = 0.879, η^2^_p_ < 0.01	*F*(1, 31) = 0.74, *p* = 0.396, η^2^_p_ = 0.02
SOA × previous congruency	*F*(1, 31) < 0.01, *p* = 0.960, η^2^_p_ < 0.01	*F*(1, 31) = 0.10, *p* = 0.751, η^2^_p_ < 0.01
Cue type × previous congruency	*F*(1, 31) = 1.50, *p* = 0.230, η^2^_p_ = 0.05	*F*(1, 31) = 3.42, *p* = 0.074, η^2^_p_ = 0.10
SOA × current congruency	*F*(1, 31) = 0.40, *p* = 0.531, η^2^_p_ = 0.01	*F*(1, 31) = 0.54, *p* = 0.466, η^2^_p_ = 0.02
Cue type × current congruency	*F*(1, 31) = 0.80, *p* = 0.378, η^2^_p_ = 0.03	*F*(1, 31) = 3.36, *p* = 0.077, η^2^_p_ = 0.10
Previous congruency × current congruency	*F*(1, 31) = 0.07, *p* = 0.791, η^2^_p_ < 0.01	*F*(1, 31) = 0.20, *p* = 0.662, η^2^_p_ < 0.01
SOA × cue type × previous congruency	*F*(1, 31) = 0.25, *p* = 0.618, η^2^_p_ < 0.01	*F*(1, 31) = 1.23, *p* = 0.277, η^2^_p_ = 0.04
SOA × cue type × current congruency	*F*(1, 31) = 0.22, *p* = 0.646, η^2^_p_ < 0.01	*F*(1, 31) = 0.19, p = 0.662, η^2^_p_ < 0.01
SOA × previous congruency × current congruency	*F*(1, 31) = 1.33, *p* = 0.258, η^2^_p_ = 0.04	*F*(1, 31) = 0.03, *p* = 0.869, η^2^_p_ < 0.01
Cue type × previous congruency × current congruency	*F*(1, 31) < 0.01, *p* = 0.973, η^2^_p_ < 0.01	*F*(1, 31) = 0.20, *p* = 0.661, η^2^_p_ < 0.01
SOA × cue type × previous congruency × current congruency	*F*(1, 31) = 0.18, *p* = 0.673, η^2^_p_ < 0.01	***F*(1, 31) = 5.85, *p* = 0.031, η**^**2**^_**p**_ **= 0.14**

*Bolded results are significant at *p* < 0.05.

#### 3.2.2 Error percentage

**Figure 2:**
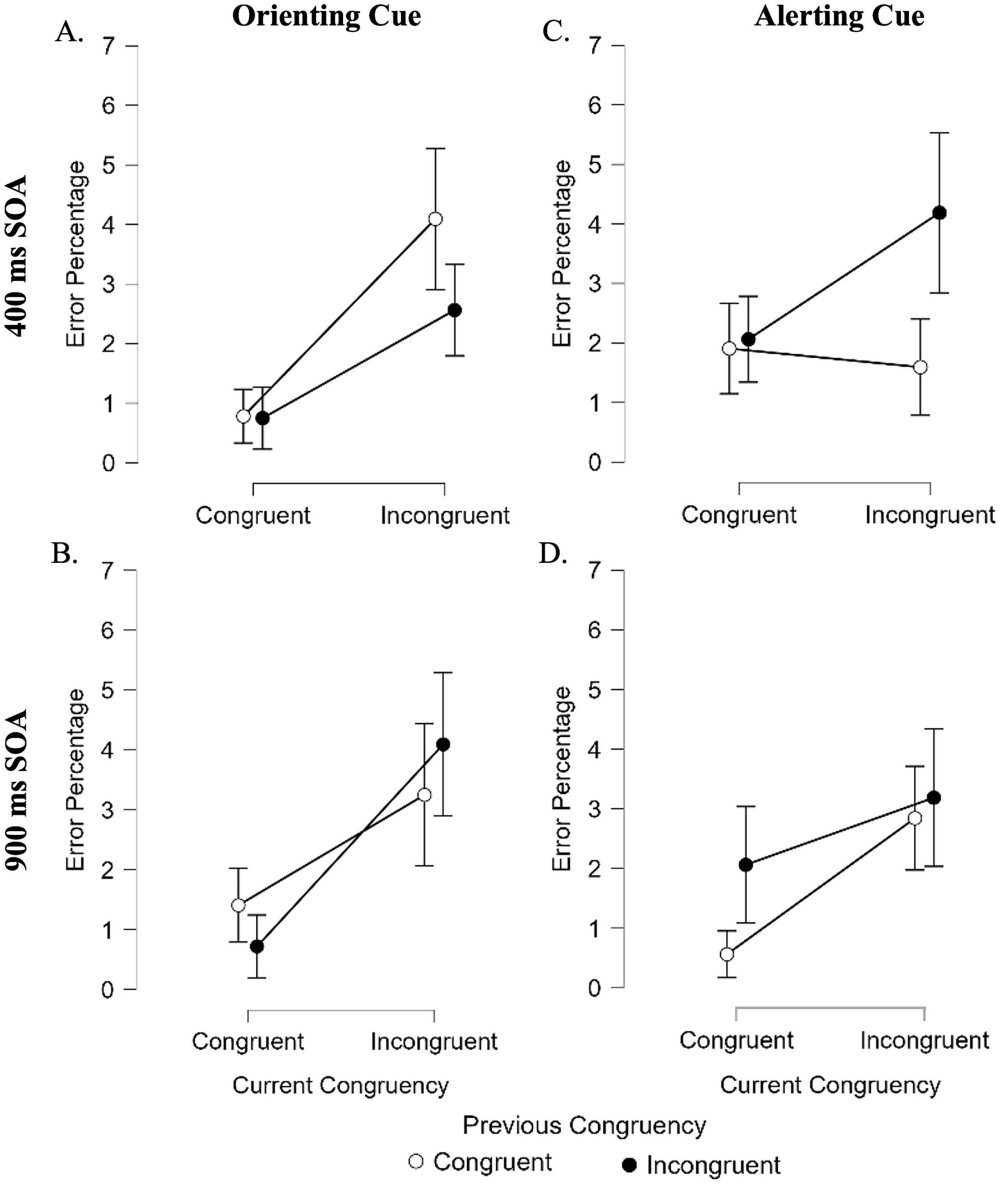
Error percentage illustrating the four-way interaction among stimulus onset asynchrony (SOA; 400 vs. 900 ms), cue type (orienting vs. alerting), previous-trial congruency, and current-trial congruency. Graphs **(A)** and **(B)** show performance following orienting cues at 400 ms and 900 ms SOAs, respectively, while Panels **(C)** and **(D)** show performance following alerting cues at 400 ms and 900 ms SOAs, respectively. Error bars represent ±1 standard error of the mean.

The full model results are reported in Table 3. The main effect of current congruency was significant, with more errors occurring on incongruent (3.2%) than congruent trials (1.3%). The four-way interaction between SOA, cue type, previous congruency, and current congruency was also significant. As illustrated in Figure 2, the typical sequential modulation of congruency (i.e., smaller executive control effects following an incongruent trial) was reversed (i.e., larger executive control effects were observed following an incongruent trial) when an alerting cue was paired with a 400-ms SOA, and when an orienting cue was paired with a 900-ms SOA.

## 4 Discussion

The ANT is a valuable tool for assessing the efficiency of different attention subsystems. While most versions rely on visual cues and stimuli, auditory adaptations are essential for extending the ANT framework to visually impaired populations and for advancing our understanding of attention in the auditory domain more broadly. Several efforts have been made to design auditory ANTs (Johnston et al., 2019; Roberts et al., 2006; Spagna et al., 2015). The present study builds on this work by examining how SOA influences auditory attention.

The auditory ANT produced the expected executive control effect: participants responded faster and more accurately when the target tone was followed by a flanker tone of the same duration, marking a congruent trial, than when target and flanker durations mismatched, marking an incongruent trial. This finding replicates prior work showing that duration discrimination tasks reliably elicit executive control effects in the auditory domain (LaCroix and Sebranek, 2025; Spagna et al., 2015). It also adds to the growing evidence that a range of tasks can effectively engage executive control in the auditory modality (Johnston et al., 2019; Petley et al., 2025; Roberts et al., 2006).

The auditory ANT also produced the expected orienting effect: responses were faster following the orienting cue (100-ms pure tone + pink noise) than the alerting cue (100-ms pink noise). This finding aligns with experiments showing that frequency cues paired with a duration discrimination task more effectively engaged auditory orienting than location cues paired with a pitch discrimination task (Johnston et al., 2019; Roberts et al., 2006; Spagna et al., 2015). Similar evidence comes from LaCroix and Sebranek (2025), who demonstrated that frequency cues embedded in duration discrimination tasks also provide a sensitive measure of orienting efficiency. While the precise mechanism driving this pattern of findings is not fully understood, Spagna et al. proposed that the tonotopic organization of the cochlea makes frequency processing more direct and efficient than processing spatial information, which relies on interaural differences.

The alerting effect, better performance on alerting cue than no cue trials, was not elicited in this experiment. This finding contrasts work using auditory only tasks, but also cross-modal attention studies where an auditory cue is paired with a visual target (Callejas et al., 2005; Han and Proctor, 2022; Ishigami et al., 2016; Johnston et al., 2019; McCormick and Christie, 2025; Roberts et al., 2006; Spagna et al., 2015; Weinbach and Henik, 2012). Our use of a pink noise burst to cue the alerting system is a key distinction from previous auditory ANTs, including Spagna et al.’s (2015) ANT-AF, which employed a complex two-tone cue. It is therefore possible that noise is less effective at inducing phasic alertness, since its broad spectral profile lacks a distinct tonal feature that can prime the auditory system. Yet previous work has successfully used noise to cue the alerting system (LaCroix and Sebranek, 2025). Notably, in LaCroix and Sebranek’s study, while the alerting effect, faster responses on noise-cued trials than on no cue trials, was present across all trials, split-half analyses showed that this effect was more stable in the second half of the task than in the first. Thus, it is also possible that the alerting system may become more responsive to cueing as the task progresses and fatigue increases; something that may not have been captured with this experiment.

While the alerting effect itself was not significant, alerting attention did interact with executive control: a smaller executive control effect was observed after an alerting cue and a larger executive control effect when no cue was presented, suggesting that the noise cue was not inert. This pattern contrasts with findings in the visual modality, where alerting cues typically enlarge the executive control effect (e.g., Fan et al., 2002; Schneider, 2018). Yet, these results align with other auditory modality findings (LaCroix and Sebranek, 2025; Spagna et al., 2015). One possible explanation is that temporal preparation is more strongly engaged in the auditory modality. By focusing attention on the expected moment of target onset, the alerting cue may make the first stimulus after the cue (the target tone) more salient than the following flanker. This increased saliency could help participants resolve conflict on incongruent trials, bringing their performance closer to that of congruent trials and thereby reducing the overall executive control effect. This same pattern may not emerge in the visual modality as the target and flanker stimuli (i.e., the five arrows) are presented together rather than being temporally staggered. Future research could test this account by requiring participants to discriminate the duration of the second tone rather than the first. Based on our hypothesis, flipping the temporal positions of the target and flanker stimuli would reverse the direction of the alertness-congruency interaction, leading to a larger executive control effect following the alerting cue. A parallel design in the visual modality, where flanker words were presented prior to the onset of the target word, showed an enlarged executive control effect following a warning signal (Fischer et al., 2012), which aligns with our hypothesis regarding the auditory modality.

Error percentages in the orienting model were modulated by SOA, cue type, and trial sequence, such that the type of cue and SOA affected the previous congruency by current congruency interaction. Prior work consistently shows that the executive control effect (the performance difference between incongruent and congruent trials) is typically *smaller* following an incongruent trial and *larger* following a congruent trial (congruency sequence effect, or CSE, e.g., Egner, 2007). We observed this expected pattern when the alerting cue was paired with a 900 ms SOA (Figure 2D) and when the orienting cue was paired with a 400 ms SOA (Figure 2A). In contrast, the reverse pattern (a *larger* executive control effect following an incongruent trial and *smaller* effect following a congruent trial) emerged when the alerting cue was paired with a 400 ms SOA (Figure 2C) and the orienting cue with a 900 ms SOA (Figure 2B). If the reversal in this interaction does not reflect cue failure, but rather the point at which each cue type is most effective, our results suggest that alerting acts over shorter timescales and orienting over longer timescales, as these are the SOAs where the expected pattern reversed. This interpretation aligns with prior work showing that phasic alertness in the visual modality is strongest at short SOAs (<500 ms) and with Spagna et al.’s (2015) argument that auditory pitch cues require more processing time than visual orienting cues. Further research is needed to clarify how SOA modulates auditory attentional subsystems, particularly because the reaction time results did not parallel the error patterns.

A notable proportion of participants (~30%) were excluded due to low accuracy, which likely reflects insufficient familiarization with the short-long duration distinction, a well-documented challenge in the temporal discrimination literature (Kopec and Brody, 2010). In the present study, participants heard only one example of each tone and then proceeded through eight practice trials; although instructions were reiterated, we did not assess practice accuracy before advancing to the experimental block. We therefore recommend that researchers implementing temporal discrimination tasks include more extensive familiarization and require participants to meet an accuracy criterion (e.g., ≥80%) before beginning experimental trials. This is the approach we use when working with clinical populations (e.g., people with aphasia), where participants repeat the training phase as needed to ensure adequate learning of the reference durations. Adopting a similar criterion in future research—whether with clinical or neurotypical samples—would help ensure task comprehension and likely reduce performance-related attrition.

In short, our findings add to the growing evidence that auditory versions of the ANT are effective tools for studying the attentional subsystems—particularly orienting and executive control. Beyond replicating the established orienting and executive control effects, the present study extends research on the auditory ANT by examining how SOA influences performance. Our findings indicate that auditory alerting and orienting may operate on different time scales, with alerting benefits emerging at shorter SOAs and orienting benefits at longer ones. The present work also lays the foundation for future research aimed at further characterizing attention in the auditory modality.

## Data Availability

The datasets presented in this study can be found in online repositories. The names of the repository/repositories and accession number(s) can be found at: https://osf.io/46f2w/?view_only=560473369b554de382cd59f818df682e.
